# Modeling a session of subject-system interaction in a wireless communication infrastructure with a mixed resource

**DOI:** 10.1371/journal.pone.0271536

**Published:** 2022-07-18

**Authors:** Viacheslav Kovtun, Ivan Izonin, Michal Gregus

**Affiliations:** 1 Department of Computer Control Systems, Vinnytsia National Technical University, Vinnytsia, Ukraine; 2 Department of Artificial Intelligence, Lviv Polytechnic National University, Lviv, Ukraine; 3 Faculty of Management, Comenius University in Bratislava, Bratislava, Slovakia; Norfolk State University, UNITED STATES

## Abstract

The article examines the subject-system interaction session, where the system is understood as a base station, and the subject is understood as a mobile communication device. The peculiarity of the study is taking into account the phenomenon relevant to modern communication infrastructures, which is that the base station supports the division of information traffic into a subspace of guaranteed personalized traffic and a subspace of general-purpose traffic. The study considers a highly critical empirical emergency when the general-purpose traffic subspace may cease to be available at any time. The presented mathematical apparatus describes the impact of such an emergency on the active communication sessions supported by the system in receiving new incoming requests of increasing intensity. To characterize this emergency situation, expressions adapted for practical application are presented to calculate such qualitative parameters as the probability of stability, the probability of failure, and unavailability.

## 1 Introduction

In today’s world, the development of telecommunications technology is outpacing space technology due to the ever-growing audience of users of mobile communication systems and their growing expectations for the quality of services and resources of wireless communication [1,2]. To avoid the depletion of limited radio resources used by modern mobile communication technologies, international standardization committees are constantly developing existing and developing new specifications governing the operation of communication networks, especially the fifth generation (5G) [3–5]. To improve the quality of customer service, such wireless communication networks are increasingly becoming integrators of both existing and promising radio access technologies. This trend causes a constant update of the list of current research problems because prompt access to information is the primary need of modern humans.

In addition to mobile access services, which provide active traffic of information made by people for people, modern mobile technologies provide information support for many applications of the Internet of Things, based on the principle of machine-to-machine interaction, and implement software-oriented networks and cloud services. It should also be noted that within the Internet of Things concept, there is a growing gap between applications focused on serving specific people and social entities and applications focused on serving industrial scenarios of the Industrial Internet. Such a variety of services and applications makes it essential to ensure the efficient operation of fifth-generation communication networks. Improving the quality of service in modern mobile communication systems requires a coordinated interaction of cellular radio access technologies with a family of WiFi local access technologies that use an unlicensed frequency spectrum. This circumstance causes frequent failures and outages in active processes of information interaction based on such a multi-technological basis [6–8].

Thus, the study of the process of subject-system interaction, which is implemented in a multi-tech communication infrastructure, where personalized traffic intersects with general-purpose traffic, is an actual scientific problem.

## 2 State-of-the-Art

Known areas of research on the analysis of systems with different mixed types of traffic in a single technological and communication environment of generation 5G focus either on the hardware-technological approach to the description of the control mechanism [9–13] or on the software concept of implementation of the latter [7–14]. It should be noted that the second approach is much more popular. It is due to the representation of the studied process in a mathematical model. The initial parameters that researchers obtain simply result from censored monitoring of the existing equipment that is operated. Accordingly, there are simply no significant and a priori irreversible financial investments that are inevitable for the first approach if the second approach is chosen.

Among the many works that study the functioning of communication systems with different mixed types of traffic, special attention should be paid to the promising direction in which this process is modeled in the paradigm of queuing theory [7–12]. Important for the construction of such models is the choice of types and characteristics of the distribution laws of stochastic controlled parameters, which characterize the processes of receiving incoming requests and service of received requests, respectively. Much attention is also stressed to the need to ensure a rationally sufficient complexity of the resulting queuing model. Choosing an algorithm for calculating the parameters of complex models of this class, even in the form of their approximation, is a task of high computational complexity.

That is why the works [10–13] describe the classic queuing systems, in which the received request is processed by precisely one service device. However, if the functioning of communication systems with different mixed types of traffic is studied, such an approximation is unacceptable. To correctly describe such a process, it is necessary to consider both the course of service of the received request and the resources allocated by the system to support it. Naturally, the volume of this resource is a random variable with some distribution law. However, in [14–18], a multilinear queuing system with a similar structure is considered, but these studies ignore the fact that different levels of reliability characterize general-purpose resources and personalized resources shared to support the received request. This fact must be reflected in the state space of the queuing system [19–23], which was not done in the above investigations.

Separately, we note the research direction of analyzing the process of functioning communication systems with different mixed types of traffic [8–16]. The results of such studies are summarized in the form of analytically determined metrics of qualitative indicators, calculating which can assess the effectiveness of the profile application of specific instances of communication systems. Suppose we focus on works where the study of this process takes place in the paradigm of the mathematical apparatus of queuing systems [14–22]. In that case, it can be argued that the authors either focus on different laws of distribution and service of incoming information requests or focus on formalizing the calculation algorithm’s characteristic parameters of the obtained stochastic models. Thus in the mentioned research, classical queuing systems in which the service of the accepted information request is carried out exclusively by one device with the allocation of the fixed volume of system resources act as the mathematical basis. These studies ignore the stochastic nature of the just mentioned parameter to simplify the resulting mathematical model analytically. The amount of system resources involved in servicing the information request in the information environment of modern base stations is dynamic rather than static, so this parameter should be part of the relevant mathematical model as a stochastic rather than deterministic characteristic. Moreover, considering that the focused channels of subject-system information interaction in the 5G infrastructure are affected by various types of interference, we, given the stochastic parameter of the system resources for information request service, should consider the queuing system to operate in failure and recovery modes according to a certain Markov process. Based on this assumption, we consider representative of the characteristics of the process of communication systems with different mixed types of compact traffic metrics in the parameter that characterizes the ability of the relevant queuing system to recover, the parameter that characterizes the probability of failure in such a system and the parameter that characterizes the availability of such a system.

Thus, the ***object*** of study is the process of functioning a communication system with different mixed types of traffic.

The ***subject*** of research is the mathematical apparatus of queuing systems for building a model of the object of study and the apparatus of mathematical statistics and probability theory to analyze the results of experiments.

The research ***aims*** to formalize symmetric metrics for evaluating the performance of a communication system that supports sessions of subject-system interaction, using both personalized guaranteed resources and general-purpose resources.

## 3 Materials and methods

### 3.1 Statement of research

We describe a balanced session of subject-system interaction in the mathematical apparatus of a multiline queuing system. Suppose that the processing of each received input request in such a system is carried out by the service apparatus, for the operation of which a resource is allocated, the volume of which is characterized by a stochastic parameter with a certain distribution law [24]. Let resource management be characterized by the Markov process of "Death and Recovery" *X*(*t*) [24,25]. Accordingly, the resource of the queuing system summarizes the stable and unstable components. In the context of the object of study, the stable component will mean the total pool of values of personal traffic volumes guaranteed to be reserved for each registered person/entity. Accordingly, an unstable component is the amount of general-purpose traffic that each registered person/entity can claim.

**Fig 1:**
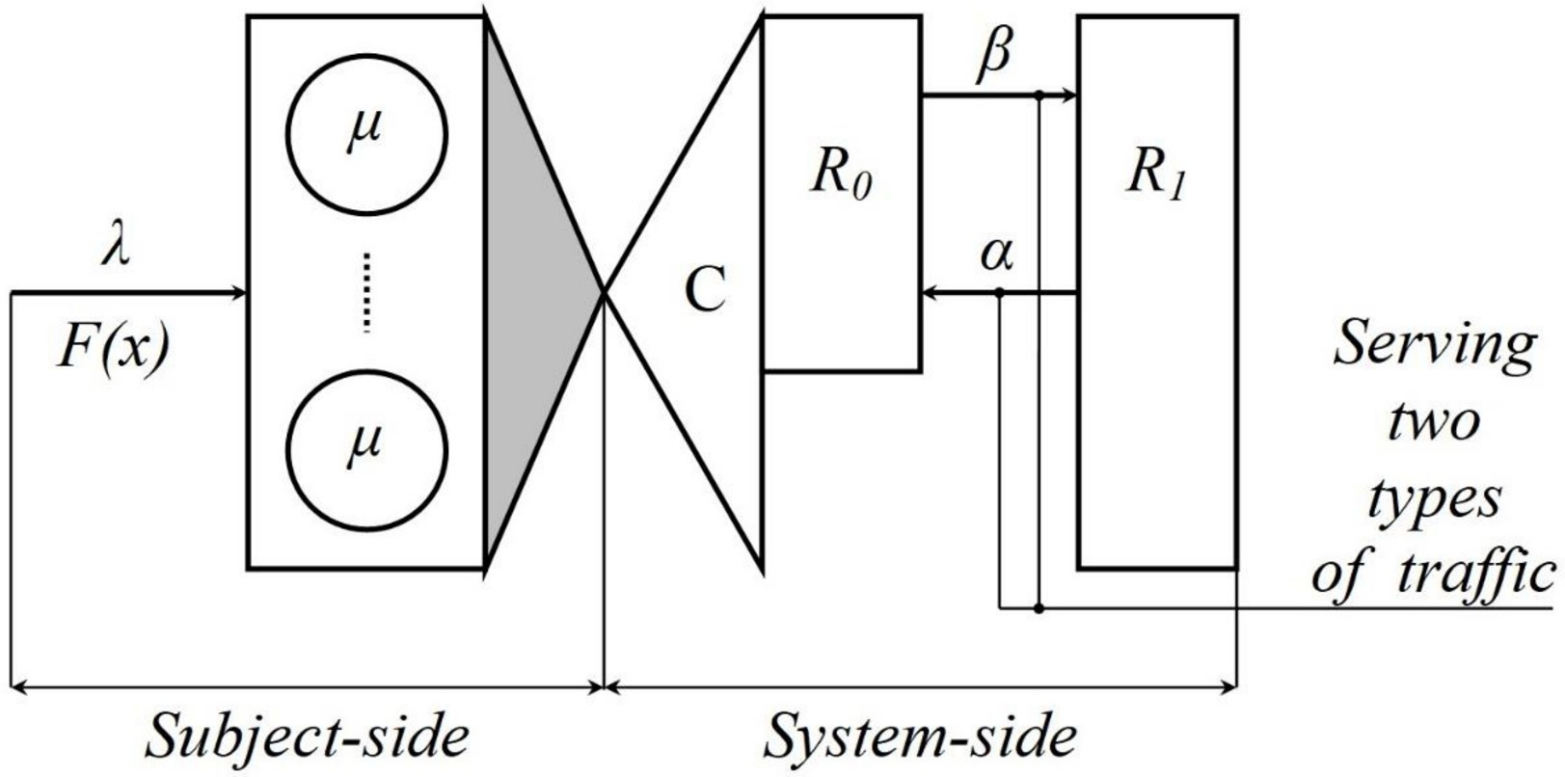
Scheme of the queuing system as a model of a balanced session of subject-system interaction.

Let the presented in Fig 1 a *C*-line queuing system direct the Poisson flow of incoming requests with intensity *λ*. The operation of servicing received requests is characterized by the parameter *μ*, distributed exponentially. At any time, such a system can be in one of two states, characterized by the binary parameter *s* ∈ {0,1}.

The transition of type *s* = 1 → *s* = 0 corresponds to the readiness mode to receive an incoming request and is characterized by intensity *α*. The transition of type *s* = 0 → *s* = 1 corresponds to the mode of loss (unpreparedness to receive) of an incoming request and is characterized by intensity *β*. When the system is in the state *s* = 1, a resource of the volume of *R*_1_ is available to service the received requests, and when the system is in the state *s* = 0, a resource of the volume of *R*_0_, *R*_0_ < *R*_1_, is available to service the received requests.

If at the time of receipt of the incoming request in the system, there are no free service devices or no free resources, the volume of which is characterized by the distribution function *F*(*x*), the incoming request is lost. In addition, if the system transitions *s* = 1 → *s* = 0 and there are *i* = *n* requests in the system for which the reserved amount of resources is $r=\sum_{i=1}^{b}r_{i}$ *r* > *R*_0_, the selected received requests will be lost. To choose which received request will be lost, two "exclusion rules" seem rational:

- *ER*1. Exclude the last received request;
- *ER*2. We exclude the one from the received requests for which the largest amount of resources is reserved.

We assume that by selecting one of the exclusion rules, the management mechanism will iteratively apply it, excluding the received requests, which will indicate the selected rule until the condition *r* ≤ *R*_0_ is not met. Note that when applying the rule *ER*1, the received request with the index *n* will always be lost *n*.

The same condition can be extended to the rule *ER*2, if at the beginning of each iteration of the procedure of exclusion of received request to re-index the set of received requests in ascending order of the value of $r_{i}\forall i=\bar{1,n}$. However, if the distribution function *F*(*x*) is not exponential, then the implementation of re-indexing will be accompanied by additional complications. Because of this circumstance, if the studied system will make the transition *s* = 1 → *s* = 0, the management mechanism for the implementation of the procedure of exclusion of received requests will apply the rule.

### 3.2 Mathematical model of the studied process

Define the Markov process of resource management in the studied queuing system as $X\left(t\right)=\left(n\left(t\right),\bar{r_{1}\left(t\right),r_{n}\left(t\right)},s\left(t\right)\right)$, where *n*(*t*) is a stochastic parameter that characterizes the number of received requests in the system; $\bar{r_{1}\left(t\right),r_{n}\left(t\right)}$ is the set of stochastic values that characterize the amount of resource reserved for the corresponding received the request; *s*(*t*) is a stochastic parameter that characterizes the state in which the system is at time *t*.

Taking into account the material of Subsection 3.1, we present the stochastic process in terms of the theory of Markov chains as

$$X\left(t\right)=\left\{\left(n,\bar{r_{1},r_{n}},s\right):0\leq n\leq C,s\in \left\{0,1\right\},r=\sum_{i=1}^{n}r_{i}\leq R_{s}\right\}.$$ (1)

Next, we will use the following notation:

$$p_{n}\left(\bar{r_{1},r_{n}}\right)=P\left\{n\left(t\right)=n,\bar{r_{1}\left(t\right),r_{n}\left(t\right)}=\bar{r_{1},r_{n}},s\left(t\right)=1\right\},$$ (2)

$$q_{n}\left(\bar{r_{1},r_{n}}\right)=P\left\{n\left(t\right)=n,\bar{r_{1}\left(t\right),r_{n}\left(t\right)}=\bar{r_{1},r_{n}},s\left(t\right)=0\right\}.$$ (3)

Let the distribution function *F*(*x*), which characterizes the distribution of the stochastic parameter of the volume of resources reserved for received requests, be continuous with a probability density *f*(*x*). Taking into account this, as well as the fact that in the transition, *s* = 1 → *s* = 0 the management mechanism works by the rule *ER*1, based on expressions (2), (3), we form a system of equilibrium equations for the Markov process defined by expression (1):

$$\begin{array}{c} p_{0}\left(\alpha +\lambda F\left(R_{1}\right)\right)=\beta q_{0}+\mu \int_{0}^{R_{1}}p_{1}\left(x\right)dx;\\ q_{0}\left(\beta +\lambda F\left(R_{0}\right)\right)=\alpha \left(\sum_{k=1}^{C}\int_{\begin{array}{l} x.\leq R_{1},\\ x_{1}\geq R_{0} \end{array}}p_{k}\left(dx_{1},\ldots ,dx_{k}\right)+p_{0}\right)+\mu \int_{0}^{R_{0}}q_{1}\left(x\right)dx;\\ \begin{array}{l} \left(\alpha +n\mu +\lambda F\left(R_{1}-x.\right)\right)p_{n}\left(\bar{r_{1},r_{n}}\right)=\mu \sum_{i=0}^{n}\int_{0}^{R_{1}-r.}p_{n+1}\left(\bar{r_{1},r_{i}},x,\bar{r_{i+1},r_{n}}\right)+\\ +\beta q_{n}\left(\bar{r_{1},r_{n}}\right)+\lambda f\left(r_{n}\right)p_{n+1}\left(\bar{r_{1},r_{n-1}}\right),\;n=\bar{1,C-1},\;r\leq R_{1}; \end{array}\\ \begin{array}{l} \left(\lambda F\left(\beta +n\mu +R_{0}-x.\right)\right)q_{n}\left(\bar{r_{1},r_{n}}\right)=\\ =\mu \sum_{i=0}^{n}\int_{0}^{R_{0}-r.}q_{n+1}\left(\bar{r_{1},r_{i}},x,\bar{r_{i+1},r_{n}}\right)dx+\lambda f\left(r_{n}\right)q_{n-1}\left(\bar{r_{1},r_{n-1}}\right)+\\ +\alpha \left(p_{n}\left(\bar{r_{1},r_{n}}\right)+\sum_{k=n+1}^{C}\int_{\begin{array}{l} \bar{x}\leq R_{1}-r.\\ x_{1}+r\geq R_{0} \end{array}}p_{k}\left({\bar{r_{1},r}}_{n},dx_{1},\ldots ,dx_{k-n}\right)\right), \end{array}\\ n=\bar{1,C-1},r\leq R_{0};\\ \left(\alpha +\mu C\right)p_{C}\left(\bar{r_{1},r_{C}}\right)=\beta q_{C}\left(\bar{r_{1},r_{C}}\right)+\lambda f\left(r_{C}\right)p_{C-1}\left(\bar{r_{1},r_{C-1}}\right),r.\leq R_{0};\\ \left(\beta +\mu C\right)q_{C}\left(\bar{r_{1},r_{C}}\right)=\alpha p_{k}\left(\bar{r_{1},r_{C}}\right)+\lambda f\left(r_{C}\right)q_{C-1}\left(\bar{r_{1},r_{C-1}}\right),r.\leq R_{0}. \end{array}$$ (4)

We will introduce a metric of indicators to assess the quality of a balanced session of subject-system interaction: {*P*,*Q*,*B*}, where:

- parameter *P*, or the probability of stability, which characterizes the probability that, as a result of the transition *s* = 0 → *s* = 1 no received request will be lost: $P\left(X\left(t\right)=\left(n,\bar{r_{1},r_{n}},0\right)\left|X\left(t-\Delta \right)=\left(n,\bar{r_{1},r_{n}},1\right)\right.\right)$:

$$P=\sum_{n=0}^{C}\int_{r\leq R_{0}}\frac{\alpha p_{n}\left(r\right)}{\alpha +n\mu +\lambda F\left(R_{1}-r.\right)}dr;$$ (5)

- parameter *Q*, or the probability of failure, which characterizes the probability that as a result of the transition *s* = 0 → *s* = 1 at least one received request will be lost: $P\left(X\left(t\right)=\left(n,\bar{r_{1},r_{n}},0\right)\left|X\left(t-\Delta \right)=\left(m,\bar{r_{1},r_{m}},1\right)\right.,n>m\right)$:

$$Q=\sum_{n=0}^{C}\int_{\bar{r}>R_{0}}\frac{\beta q_{n}\left(r\right)}{\beta +n\mu +\lambda F\left(R_{1}-r.\right)}dr.$$ (6)

- parameter *B*, or the probability of unavailability, which describes the probability of termination for non-acceptance of the service from the pool of subscribers with guaranteed personalized traffic due to an emergency:

B=∫x≤R1pCdxi+∫x≤R0qcdxi+∑n=0C−1∫x≤R1pcdxi××1−FR1−r+∫x≤R0qdCxi1−FR0−r,i=1,C¯.

The system of equilibrium Eq (4) and the metric of qualitative indicators (5), and (6) unambiguously characterize the studied process in a continuous-time. However, obtaining an analytical solution to the system of equilibrium equations of the form (4) for an arbitrary distribution function *F*(*x*) seems doubtful. This fact leads to the continuation of the investigation of the stochastic process in a discrete distribution of resources.

Let the parameter *p*(*x*) characterize the probability that the input request sent to the system will be received with the allocation to maintain the number of resources equal to *x*. Considering the parameter *p*(*x*), we rewrite the system of equilibrium Eq (4). As a result, we will receive:

$$\begin{array}{c} p_{0}\left(\alpha +\lambda F\left(R_{1}\right)\right)=\beta q_{0}+\mu \sum_{i=0}^{R_{1}}q_{i}\left(i\right)\\ q_{0}\left(\beta +\lambda F\left(R_{0}\right)\right)=\alpha \left(p_{0}+\sum_{k=1}^{C}\sum_{\begin{array}{c} r.\leq R_{1},\\ r_{1}>R_{0} \end{array}}p_{k}\left(\bar{r_{1},r_{k}}\right)\right)+\mu \sum_{i-0}^{R_{0}}q_{1}\left(i\right);\\ \begin{array}{l} \left(\beta +n\mu +\lambda F\left(R_{0}-r.\right)\right)q_{n}\left(\bar{r_{1},r_{n}}\right)=\\ =\alpha \left(p_{n}\left(\bar{r_{1},r_{n}}\right)+\sum_{k=n+1}^{C}\sum_{\begin{array}{c} R_{0}-r_{n+1}<\\ <\sum_{i=1}^{n}r_{i}\leq R_{0},\\ r\leq R_{1} \end{array}}p_{k}\left(\bar{r_{1},r_{n}},\bar{r_{n+1},r_{k}}\right)\right)+\\ +\mu \sum_{i=1}^{n+1}\sum_{r_{i}=0}q_{n+1}\left(\bar{r_{1},r_{i}},\bar{r_{i+1},r_{n}}\right)+\lambda p\left(r_{n}\right)q_{n-1}\left(\bar{r_{1},r_{n-1}}\right), \end{array}\\ n=\bar{1,C-1};\\ \begin{array}{l} \left(\alpha +n\mu +\lambda F\left(R_{1}-r.\right)\right)p_{n}\left(\bar{r_{1},r_{n}}\right)=\beta q_{n}\left(\bar{r_{1},r_{n}}\right)+\\ +\lambda p\left(r_{n}\right)p_{n-1}\left(\bar{r_{1},r_{n-1}}\right)+\mu \sum_{i=1}^{n+1}\begin{array}{l} \sum_{j=r_{i}=0}^{R_{1}-\sum_{j\neq i}r_{j}}q_{n+1}\left(\bar{r_{1},r_{i}},\bar{r_{i+1},r_{n}}\right)\\ n=\bar{1,C-1}; \end{array} \end{array}\\ \left(\alpha +\mu C\right)p_{C}\left(\bar{r_{1},r_{C}}\right)=\beta q_{n}\left(\bar{r_{1},r_{n}}\right)+\lambda f\left(r_{C}\right)p_{C-1}\left(\bar{r_{1},r_{C-1}}\right);\\ \left(\beta +\mu C\right)q_{C}\left(\bar{r_{1},r_{C}}\right)=\alpha p_{k}\left(\bar{r_{1},r_{C}}\right)+\lambda f\left(r_{C}\right)q_{C-1}\left(\bar{r_{1},r_{C-1}}\right). \end{array}$$ (7)

The lack of description of the process *X*(*t*) by the system of equilibrium equations of the form (7) is the inherent large dimension of the latter. To prevent this shortcoming, we move from the process *X*(*t*) to the Markov process *Y*(*t*) = (*n*(*t*), *r*(*t*), *s*(*t*)) with the state space

$$Y\left(t\right)=\left\{\left(n,r,s\right):0\leq n\leq C,s\in \left\{0,1\right\},r\leq R_{s}\right\}.$$ (8)

The parameter *r* used in expression (8) characterizes the total amount of reserved resources for all *n* received requests. Under this approach, the number of resources is released at the end of the service or the loss of the received request in the system is released, which is determined by the formula of conditional probability [26–29]. Considering this assumption, redefine expressions (2) and (3):

$$p_{n}\left(r\right)=P\left\{n\left(t\right)=n,r\left(t\right)=r,s\left(t\right)=1\right\},$$ (9)

$$q_{n}\left(r\right)=P\left\{n\left(t\right)=n,r\left(t\right)=r,s\left(t\right)=0\right\}.$$ (10)

Taking into account expressions (9), and (10), we proceed to the corresponding stochastic process (8) of the representation of the system of equilibrium Eq (7). As a result, we will receive:

$$\left(\alpha +\lambda F\left(R_{1}\right)\right)p_{0}\left(0\right)=\beta q_{0}\left(0\right)+\mu \sum_{j=0}^{R_{1}}p_{1}\left(j\right),$$ (11)

$$\begin{array}{l} \left(\beta +\lambda F\left(R_{1}\right)\right)q_{0}\left(0\right)=\mu \sum_{j=0}^{R_{0}}q_{1}\left(j\right)+\\ +\alpha \left(p_{0}\left(0\right)+\sum_{i=1}^{C}\sum_{j=R_{0}+1}^{R_{1}}p_{i}\left(j\right)\sum_{s=R+1}^{j}\frac{{\left(p\left(j-s\right)\right)}^{\left(i-1\right)}p\left(s\right)}{{\left(p\left(j\right)\right)}^{\left(i\right)}}\right); \end{array}$$ (12)

$$\begin{array}{l} \left(\alpha +n\mu +\lambda F\left(R_{1}-r\right)\right)p_{n}\left(r\right)=\beta q_{n}\left(r\right)+\lambda \sum_{j=0}^{r}p_{n-1}\left(r-j\right)p\left(j\right)+\\ +\mu \sum_{j=0}^{R_{1}-r}\sum_{i=1}^{n+1}\frac{p_{n+1}\left(r+j\right){\left(p\left(j\right)\right)}^{\left(i\right)}{\left(1-p\left(j\right)\right)}^{\left(n-i+1\right)}}{},\;n=\bar{1,C-1}; \end{array}$$ (13)

$$\begin{array}{l} \left(\beta +n\mu +\lambda F\left(R_{1}-r\right)\right)q_{n}\left(r\right)=\mu \sum_{j=0}^{R_{0}-r}\frac{q_{n+1}\left(r+j\right)p\left(j\right)p{\left(r\right)}^{\left(n\right)}}{{\left(p\left(r+j\right)\right)}^{\left(n+1\right)}}+\lambda \sum_{j=0}^{r}q_{n-1}\left(r-j\right)p\left(j\right)+\\ +\alpha \left(p_{n}\left(r\right)+\sum_{i=n+1}^{C}\sum_{j=R_{0}+1}^{R_{1}}p_{i}\left(j\right)\sum_{s=R_{0}-r+1}^{j-r}\frac{p\left(s\right){\left(p\left(r\right)\right)}^{\left(n\right)}{\left(p\left(j-r-s\right)\right)}^{\left(i-n-1\right)}}{{\left(p\left(j\right)\right)}^{\left(i\right)}}\right),\;n=\bar{1,C-1}; \end{array}$$ (14)

$$\left(\alpha +n\mu \right)p_{C}=\beta q_{n}\left(r\right)+\lambda \sum_{j=0}^{r}p_{C-1}\left(r-j\right)p\left(j\right),$$ (15)

$$\left(\beta +n\mu \right)q_{C}=\alpha p_{C}\left(r\right)+\lambda \sum_{j=0}^{r}q_{C-1}\left(r-j\right)p\left(j\right),$$ (16)

where (*p*(*r*))^(*n*)^ is a *n*-fold convolution, which characterizes the probability that to service *n* received requests in the system will be reserved amount of resources equal to *r*.

## 4 Results

To apply computational methods to solve the system of equilibrium Eqs (11)–(16), the latter must be presented in matrix form. To do this, we present the state space of the stochastic process *Y*(*t*) in the form of disjoint subsets:

$$Y=Y_{0}\cup Y_{1}\cup Y_{2},$$

where

$$Y_{0s}=\left\{0,s;s\in \left\{0,1\right\}\right\},$$

$$Y_{js}=\left\{\bar{\left(j,0,s\right),\left(j,R_{0},s\right)};j=\bar{1,C},s\in \left\{0,1\right\}\right\},$$

$$Y_{j2}=\left\{\bar{\left(j,R_{0}+1,1\right),\left(j,R_{1},1\right)};j=\bar{1,C}\right\},Y_{s}=\overset{C}{\underset{i=0}{\cup }}Y_{is};s\in \left\{0,1\right\}.$$

Taking into account the recorded, we define the matrix of intensities of transitions of the stochastic process *Y*(*t*) in the block-diagonal form:

$$A=\left(\begin{array}{ccc} A_{00} & \ldots & A_{02}\\ \vdots & \ddots & \\ A_{20} & \cdots & A_{22} \end{array}\right),$$ (17)

where

$$A_{00}=A_{11}=\left(\begin{array}{cccc} D_{00} & B_{00} & 0 & 0\\ F_{01} & D_{01} & \ldots & 0\\ 0 & \vdots & \ddots & B_{0C-1}\\ 0 & 0 & L_{0C} & D_{0C} \end{array}\right),A_{01}=\text{diag}\left(\beta \right),A_{02}=\left(0\right),A_{10}=\text{diag}\left(\alpha \right),$$

$$A_{12}=\left(\begin{array}{ccc} B_{20} & 0 & 0\\ 0 & \ddots & 0\\ \vdots & & B_{2C-1}\\ 0 & 0 & 0 \end{array}\right),A_{20}=\left\{\begin{array}{c} \begin{array}{l} \alpha \frac{{\left(p\left(r\right)\right)}^{\left(n\right)}}{{\left(p\left(j\right)\right)}^{\left(i\right)}}\sum_{s=R_{0}-r+1}^{j-r}p\left(s\right){\left(p\left(j-r-s\right)\right)}^{\left(i-n-1\right)}\forall \\ \forall r=\vec{0,R_{0}};n=\vec{0,i};j=\bar{R_{0}+1,R_{1}};i=\bar{0,C}; \end{array}\\ 0,\;\text{otherwise,} \end{array}\right.,$$

$$A_{21}=\left(\begin{array}{cccc} F_{21} & 0 & \ldots & 0\\ 0 & & \ddots & 0\\ 0 & 0 & F_{2C} & 0 \end{array}\right),A_{22}=\left(\begin{array}{cccc} D_{10} & B_{10} & 0 & 0\\ F_{11} & D_{11} & \ldots & 0\\ 0 & \vdots & \ddots & B_{1C-1}\\ 0 & 0 & F_{1C} & D_{1C} \end{array}\right),$$

$$B_{0i}=\left(\lambda p\left(t-j\right)\right),i=\bar{j,R_{0}},j=\bar{0,R_{0}},B_{1i}=\left(\lambda p\left(t-j\right)\right),i=\bar{j,R_{1}},j=\bar{0,R_{1}},$$

$$B_{2i}=\left(\lambda p\left(t-j\right)\right),i=\bar{j,R_{1}},j=\bar{0,R_{0}},F_{0i}=\left(\mu i\sum_{j=0}^{R-r}\frac{{\left(1-p\left(j\right)\right)}^{\left(n-i+1\right)}{\left(p\left(j\right)\right)}^{\left(j\right)}}{{\left(p\left(r+j\right)\right)}^{\left(n+1\right)}}\right),$$

$$i=\bar{0,j},j=\bar{0,R_{0}},F_{1i}=\left(\mu i\sum_{j=0}^{R-r}\frac{{\left(1-p\left(j\right)\right)}^{\left(n-i+1\right)}{\left(p\left(j\right)\right)}^{\left(j\right)}}{{\left(p\left(r+j\right)\right)}^{\left(n+1\right)}}\right),i=\bar{R_{0},j},j=\bar{R_{0},R_{1}};$$

$$F_{2i}=\left(\mu i\sum_{j=0}^{R-r}\frac{{\left(1-p\left(j\right)\right)}^{\left(n-i+1\right)}{\left(p\left(j\right)\right)}^{\left(j\right)}}{{\left(p\left(r+j\right)\right)}^{\left(n+1\right)}}\right),i=\bar{R_{0}+1,R_{1}},j=\bar{0,R_{0}};$$

$$D_{0i}=\text{diag}\left(\beta +n\mu -\lambda F\left(R_{0}-r\right)\right),D_{1i}=\text{diag}\left(\alpha +n\mu -\lambda F\left(R_{1}-r\right)\right),$$

$$D_{2i}=\text{diag}\left(\alpha +n\mu -\lambda F\left(R_{1}-r\right)\right).$$

The system of equilibrium Eqs (11)–(16) presented in form (17) can be solved by known computational methods, for example, iterative [26–28,30].

Consider the application of the mathematical apparatus presented in Section 3, adapted to the form represented by expression (17). We will experiment with the Huawei 5G base station. The resource in the experiment was understood as the speed of the session of the subject-system information interaction, where the system was considered a base station, and the subjects–smartphones iPhone 13 Pro. To ensure the statistical reliability of the calculation results, 1000 sessions were performed in the conditions of agglomeration of a modern metropolis at a distance of about 1 km from the base station.

For the experiment, a bandwidth of personalized use *C*_0_ with a width of 10 MHz was allocated. With the help of the LSA service, a 5 MHz wide bandwidth of general-purpose use *C*_1_−*C*_0_ was additionally allocated. Accordingly, the session speed for these frequency bands was calculated by the expression *R*_*s*_ = *C*_*s*_*V*_*s*_, where *V*_*s*_ is the spectral efficiency, which for the experiments, taking into account the equipment, its settings, and operating conditions were 4 bps/Hz. Accordingly, the amount of guaranteed personalized traffic was *R*_0_ = 40 Mbps, and the amount of general-purpose traffic was *R*_1_−*R*_0_ = 20 Mbps. The total amount of traffic *R*_1_, as a result, was 60 Mbps. Under the conditions of the experiment, one session of subject-system interaction utilizes 2 Mbps of traffic, i.e. *F*(*x*) = {0∀*x*≤2,1*x>*2}. The other average values of the characteristic parameters of the sessions of the subject-system interaction are as follows: 1/*α* = 120 c, 1/*β* = 60 c, 1/*μ* = 15 c.

**Fig 2:**
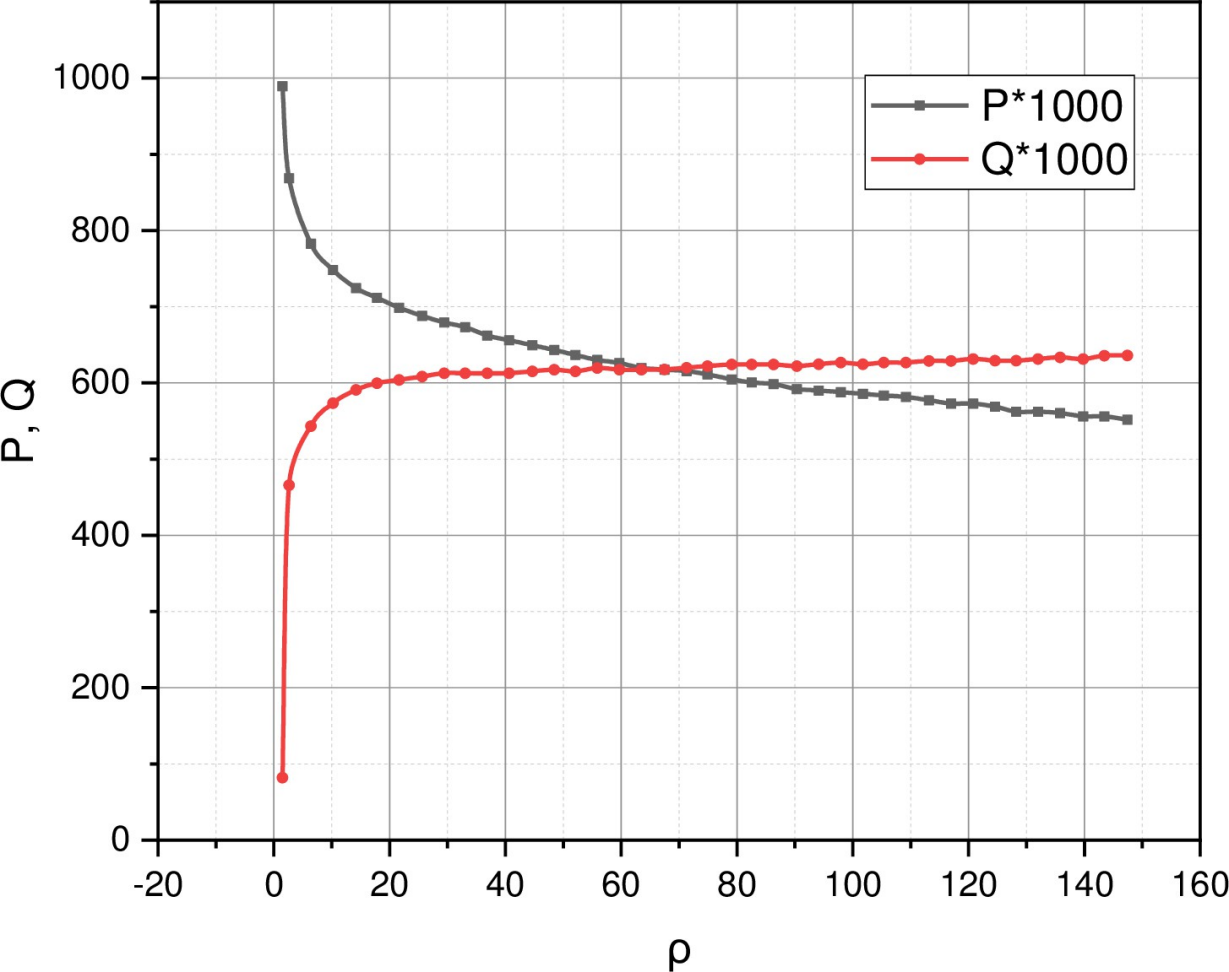
Functional dependences {*P*,*Q*} = *f*(*ρ*) calculated based on model (17) for the obtained experimental data.

The results of the experiment allowed us to calculate the functional dependencies {*P*,*Q*} = *f*(*ρ*), where the parameters *P* and *Q* are the probability of stability and the probability of failure, which are analytically characterized by expressions (5) and (6), respectively, and *ρ* is the parameter of the intensity of load determined by expression *ρ* = *λ*/*μ*. The functional dependences calculated based on the mathematical model generalized by expression (17) and the received experimental data are presented in Fig 2.

**Fig 3:**
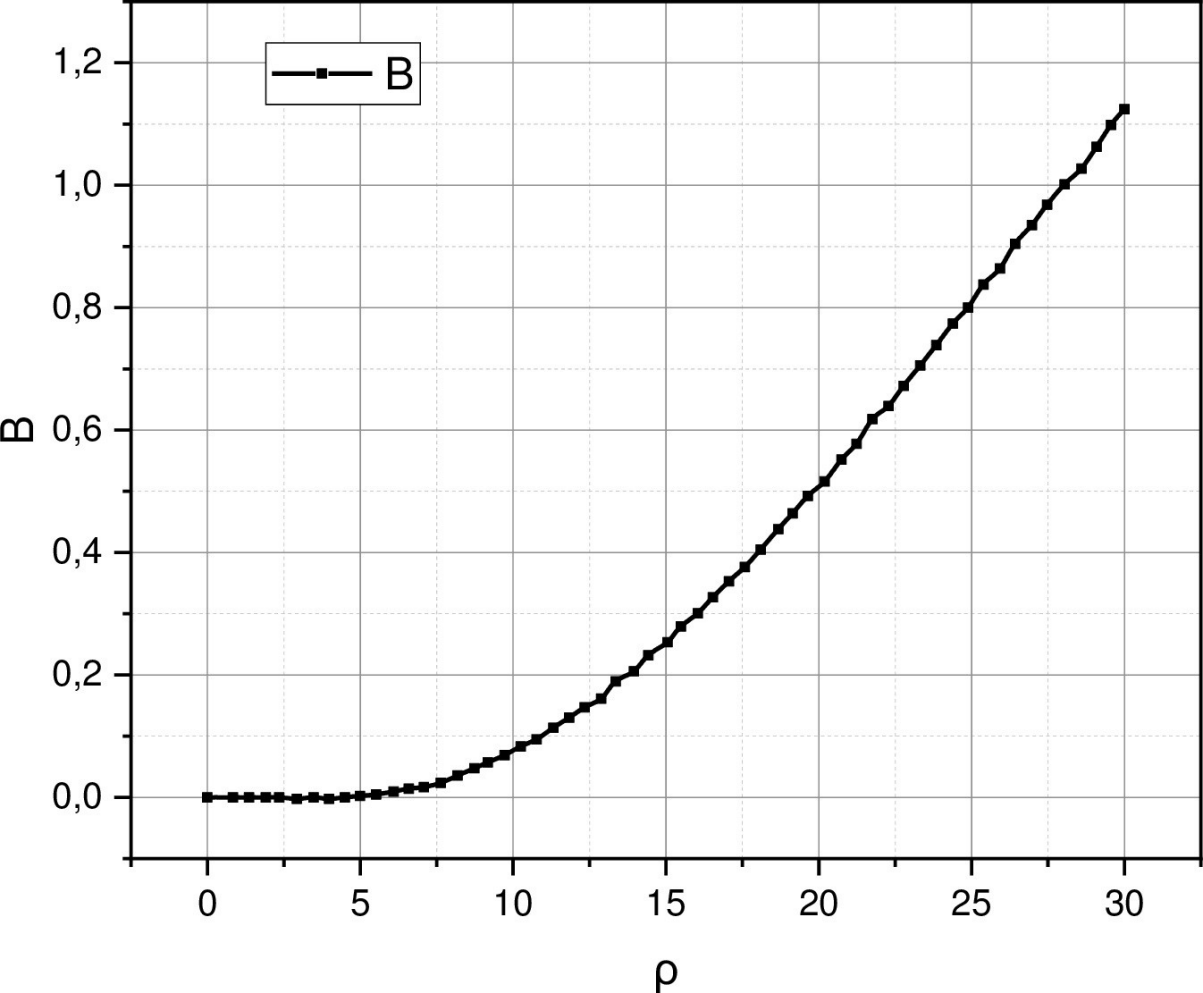
Functional dependencies *B* = *f*(*ρ*) calculated based on model (17) for the obtained experimental data.

A derivative of the qualitative indicators (5) and (6) calculated on the basic models (17) is the probability of unavailability. This qualitative characteristic is calculated based on parameters (9) and (10) determined as a result of solving the system of equilibrium Eqs (11)–(16). In the context of the study of the subject-system interaction session in a centralized wireless communication system with a mixed resource, the unavailability parameter is a significant empirical characteristic. This parameter describes the probability of termination for non-acceptance of the service from the pool of subscribers with guaranteed personalized traffic due to an emergency. Suppose the qualitative parameters describe the impact of the emergency on the already active sessions of the subject-system interaction (internal characteristics). In that case, the quality parameter describes the impact of the emergency on the ability of the base station to accept new incoming requests (external characteristics). The empirical dependence *B* = *f*(*ρ*) calculated for the studied system is presented in Fig 3.

## 5 Discussion

As shown in Fig 2, calculated functional dependencies {*P*,*Q*} = *f*(*ρ*) can draw several conclusions. First, the mathematical model (17) adequately describes the course of sessions of subject-system interaction in a properly functioning network infrastructure under study. It is seen that with increasing load, i.e., with increasing intensity of the flow of incoming requests, the probability of stability *P* begins to decrease, and the probability of failure *Q* begins to increase.

To move on to the second thesis, let’s make a small preamble. Recall that the indicator *P*(*Q*) characterizes the probability that as a result of the transition, *s* = 0 → *s* = 1 none (at least one) received request will be lost. The event when the studied network infrastructure makes the transition *s* = 0 → *s* = 1 corresponds to a situation when, for some reason, the resource of public-purpose traffic becomes unavailable, but the resource of personalized guaranteed traffic remains relevant. Returning to the calculated dependencies {*P*,*Q*} = *f*(*ρ*), we can see that even in the conditions of instantaneous transition *s* = 0 → *s* = 1, which is characteristic of Markov processes and correlates with the situation of an emergency shutdown of additional communication capacities, the probability of stability *P* and the probability of failure *Q* does not change abruptly. These dynamics indicate that until the load *ρ* reaches a certain limit load level, the base station provides support for received requests within the guaranteed traffic volume (nonlinear part of curves {*P*,*Q*} = *f*(*ρ*)), and only after crossing the load value of this level (linear part of curves {*P*,*Q*} = *f*(*ρ*)) general-purpose resource begins to be used. Based on these facts, it can be argued that the proposed metric {*P*,*Q*} is simple and effective in the task of assessing the survivability or functional safety of modern centralized wireless info-communication systems.

Third, in favor of the adequacy of the mathematical model presented in Section 3 speaks of the correctness of the modeling task statement, the rationality of the choice of the basic mathematical apparatus, and the logic of the mathematical transformations. The author foresaw the possibility of an accurate (expression (7)) or approximate (expressions (11)-(16)) description of the studied process. Representation of the original data in the obtained mathematical apparatus, embodied in the graphs in Fig 2, corresponds to the actual operation of the test equipment.

**Fig 4:**
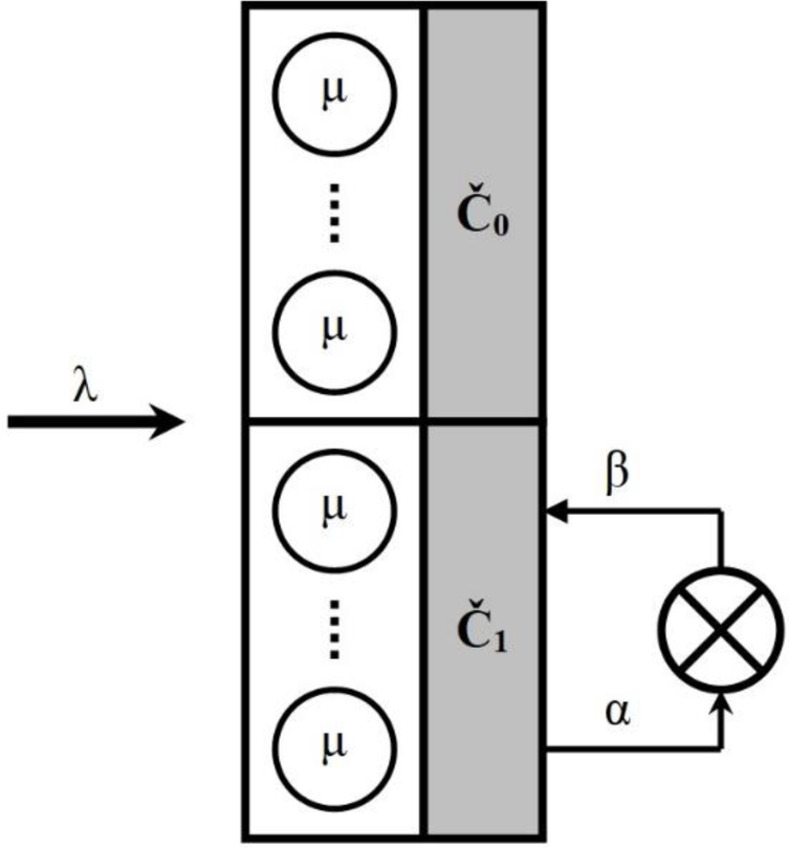
Promising scheme of improving the system presented in Fig 1.

Interpreting the presented a Fig 3 behavior of the studied system with the generalized structure presented in Fig 1, we can draw the following conclusion: if the distribution function of the mixed resource is deterministic, then the model in Fig 1 can be reduced to the form shown in Fig 4.

Two sets of service devices form such a system without resources (reliable: ${\overset{⌣}{C}}_{0}$; unreliable: ${\overset{⌣}{C}}_{1}$). Proving this thesis is a promising area of further research.

## 6 Conclusions

The article examines the subject-system interaction session, where the system is understood as a base station, and the subject is understood as a mobile communication device. The peculiarity of the study is taking into account the phenomenon relevant to modern communication infrastructures, which is that the base station supports the division of information traffic into a subspace of guaranteed personalized traffic and a subspace of general-purpose traffic. The study considers a highly critical empirical emergency situation when the general-purpose traffic subspace may cease to be available at any time.

The presented mathematical apparatus (based on a multilinear queuing system) describes the impact of such an emergency on the active communication sessions supported by the system in the conditions of receiving new incoming requests of increasing intensity. To characterize this emergency situation, expressions adapted for practical application are presented to calculate such qualitative parameters as the probability of stability, the probability of failure, and unavailability. The *first /second/* indicator characterizes the probability that as a result of disabling the subspace of public traffic, no one (at least one) who received the request *will not be /will be/* lost. The third quality parameter describes the emergency’s impact on the base station’s ability to accept *new incoming requests /external characteristics/*. The adequacy of the proposed mathematical apparatus and the rationality of the presented metrics of qualitative parameters are proved empirically. The obtained results indicate that such a metric will be especially relevant in the operational assessment of survivability or functional safety of wireless centralized network infrastructure.

In Fig 2, we can see that even in the conditions of instantaneous transition *s* = 0 → *s* = 1, which is characteristic of Markov processes and correlates with an emergency shutdown of additional communication capacities, the probability of stability *P* and the probability of failure *Q* does not change abruptly. These dynamics indicate that until the load *ρ* reaches a certain limit load level, the base station provides support for received requests within the guaranteed traffic volume (nonlinear part of curves {*P*,*Q*} = *f*(*ρ*)), and only after crossing the load value of this level (linear part of curves {*P*,*Q*} = *f*(*ρ*)) general-purpose resource begins to be used.

***Further research*** is proposed to focus on developing the mathematical apparatus presented in the article by taking into account the different options for the resource allocation function between the system-supported sessions of information interaction.

## Supporting information

S1 Data (XLS)

## References

[1] Obelovska K, Panova O, Karovič V. Performance Analysis of Wireless Local Area Network for a High-/Low-Priority Traffic Ratio at Different Numbers of Access Categories. Symmetry. 2021 Apr 15;13(4):693. DOI: 10.3390/sym13040693

[2] Droniuk I, Fedevych O. Forecasting of the trend of traffic based on Ateb-functions theory. In: 2015 Xth International Scientific and Technical Conference ‘Computer Sciences and Information Technologies’ (CSIT). 2015. p. 139–41.

[3] Yaqoob M, Gemikonakli O, Ever E. Modelling heterogeneous future wireless cellular networks: An analytical study for interaction of 5G femtocells and macro-cells. Future Generation Computer Systems. 2021 Jan;114:82–95. DOI: 10.1016/j.future.2020.07.049

[4] Tang L, Zhao G, Wang C, Zhao P, Chen Q. Queue-aware reliable embedding algorithm for 5G network slicing. Computer Networks. 2018 Dec;146:138–50.

[5] Boutiba K, Ksentini A, Brik B, Challal Y, Balla A. NRflex: Enforcing network slicing in 5G New Radio. Computer Communications. 2022 Jan;181:284–92.

[6] Xie Y, Wang S, Wang B, Xu S, Wang X, Ren J. Online algorithm for migration aware Virtualized Network Function placing and routing in dynamic 5G networks. Computer Networks. 2021 Jul;194:108115.

[7] Slalmi A, Chaibi H, Saadane R, Chehri A. Call Admission Control Optimization in 5G in Downlink Single-Cell MISO System. Procedia Computer Science. 2021;192:2502–11.

[8] Bilen T, Canberk B. Overcoming 5G ultra-density with game theory: Alpha-beta pruning aided conflict detection. Pervasive and Mobile Computing. 2020 Mar;63:101133. DOI: 10.1016/j.pmcj.2020.101133

[9] Qiao L, Li Y, Chen D, Serikawa S, Guizani M, Lv Z. A survey on 5G/6G, AI, and Robotics. Computers & Electrical Engineering. 2021 Oct;95:107372.

[10] Hovorushchenko TO. Methodology of Evaluating the Sufficiency of Information for Software Quality Assessment According to ISO 25010. J inf organ sci (Online). 2018 Jun 26;42(1):63–85.

[11] Zhou Y, Li L. The 5G communication technology-oriented intelligent building system planning and design. Computer Communications. 2020 Jul;160:402–10.

[12] Li B, Hou P, Wu H, Hou F. Optimal edge server deployment and allocation strategy in 5G ultra-dense networking environments. Pervasive and Mobile Computing. 2021 Apr;72:101312.

[13] Haile H, Grinnemo K-J, Ferlin S, Hurtig P, Brunstrom A. End-to-end congestion control approaches for high throughput and low delay in 4G/5G cellular networks. Computer Networks. 2021 Feb;186:107692. doi: 10.1016/j.comnet.2020.107692

[14] Muhammad M, Safdar GA. 5G-based V2V broadcast communications: A security perspective. Array. 2021 Sep;11:100084.

[15] Al-Ma’aitah M, Saad A, Alwadain A. Modeling of the Schemes for Organizing a Session of Person–System Interactions in the Information System for Critical Use Which Operates in a Wireless Communication Environment. Symmetry. 2021 Feb 27;13(3):391.

[16] Kovtun V, Izonin I. Study of the Operation Process of the E-Commerce Oriented Ecosystem of 5Ge Base Station, Which Supports the Functioning of Independent Virtual Network Segments. JTAER. 2021 Oct 23;16(7):2883–97.

[17] Han S. Congestion-aware WiFi offload algorithm for 5G heterogeneous wireless networks. Computer Communications. 2020 Dec;164:69–76.

[18] Bruschi R, Pajo JF, Davoli F, Lombardo C. Managing 5G network slicing and edge computing with the MATILDA telecom layer platform. Computer Networks. 2021 Jul;194:108090.

[19] Ali Z, Naz F, Javed, Qurban M, Yasir M, Jehangir S. Analysis of VoIP over Wired & Wireless Network with Implementation of QoS CBWFQ & 802.11e. IJCNIS. 2020 Feb 8;12(1):43–9.

[20] M P B, Mahadeva Swamy UB, Shrynik Jain MB. Inter Integrated WSN for Crude Oil Pipeline Monitoring. IJCNIS. 2018 Mar 8;10(3):37–51.

[21] Rao AS. Improving the Serviceability of a Prepaid Autorickshaw Counter using Queuing Model: An Optimization Approach. IJITCS. 2017 Dec 8;9(12):19–27.

[22] Randa Hammami, Kacem Yossra H, Senda Souissi, Hatem Bellaaj, Kacem Ahmed H. Weighted Priority Queuing: A New Scheduling Strategy for Web Services. IJITCS. 2017 Feb 8;9(2):11–7.

[23] Das I, Lobiyal DK, Katti CP. Queuing Effect on Multipath Routing in Mobile Ad Hoc Networks. IJIEEB. 2016 Jan 8;8(1):62–8.

[24] Alakbarov RG, Pashaev FH, Hashimov MA. Development of the Model of Dynamic Storage Distribution in Data Processing Centers. IJITCS. 2015 Apr 8;7(5):18–24. DOI: 10.5815/ijitcs.2015.05.03

[25] Dalkani H, Mojarad M, Arfaeinia H. Modelling Electricity Consumption Forecasting Using the Markov Process and Hybrid Features Selection. IJISA. 2021 Oct 8;13(5):14–23.

[26] Michael Chuma F, Godson Mwanga G. Stability Analysis of Equilibrium Points of Newcastle Disease Model of Village Chicken in the Presence of Wild Birds Reservoir. IJMSC. 2019 Apr 8;5(2):1–18.

[27] Umar Y, Bashir Ibrahim M, Department of Pure and Industrial Chemistry, Bayero University. Equilibrium and Thermodynamic Studies on Adsorption of Hexavalent Chromium from Aqueous Solution onto Low Cost Activated Carbon. IJEM. 2020 Apr 8;10(2):52–70.

[28] Hu Z, Ivashchenko M, Lyushenko L, Klyushnyk D. Artificial Neural Network Training Criterion Formulation Using Error Continuous Domain. IJMECS. 2021 Jun 8;13(3):13–22.

[29] Izonin I, Tkachenko R, Ryvak L, Zub K, Rashkevych M, Pavliuk O. Addressing Medical Diagnostics Issues: Essential Aspects of the PNN-based Approach. CEUR. 2020 Jan 12; 2753:209–218.

[30] Hu Z, Odarchenko R, Gnatyuk S, Zaliskyi M, Chaplits A, Bondar S, et al. Statistical Techniques for Detecting Cyberattacks on Computer Networks Based on an Analysis of Abnormal Traffic Behavior. IJCNIS. 2021 Dec 8;12(6):1–13.

